# Cerebrospinal fluid oxidative stress metabolites in patients with bipolar disorder and healthy controls: a longitudinal case-control study

**DOI:** 10.1038/s41398-019-0664-6

**Published:** 2019-11-28

**Authors:** Ulla Knorr, Anja Hviid Simonsen, Peter Roos, Allan Weimann, Trine Henriksen, Ellen-Margrethe Christensen, Maj Vinberg, Rie Lambæk Mikkelsen, Thomas Kirkegaard, Rasmus Nejst Jensen, Morten Akhøj, Julie Forman, Henrik Enghusen Poulsen, Steen Gregers Hasselbalch, Lars Vedel Kessing

**Affiliations:** 1 Copenhagen Affective Disorder Research Center (CADIC), Psychiatric Center Copenhagen, University of Copenhagen, Faculty of Health and Medical Sciences, Copenhagen, Denmark; 2 Danish Dementia Research Centre, section 6922, Rigshospitalet, University of Copenhagen, Faculty of Health and Medical Sciences, Copenhagen, Denmark; 3 Laboratory of Clinical Pharmacology, Rigshospitalet, University of Copenhagen, Faculty of Health and Medical Sciences, Copenhagen, Denmark; 4 Department of Clinical Pharmacology, Bispebjerg Hospital, University of Copenhagen, Faculty of Health and Medical Sciences, Copenhagen, Denmark; 5 Section of Biostatistics, Department of Public Health, University of Copenhagen, Copenhagen, Denmark

**Keywords:** Diagnostic markers, Neuroscience

## Abstract

Bipolar disorder (BD) is a mental disorder characterized by recurrent relapses of affective episodes, cognitive impairment, illness progression, and reduced life expectancy. Increased systemic oxidatively generated nucleoside damage have been found in some neurodegenerative disorders and in BD. As the first, this naturalistic prospective, longitudinal follow-up case-control study investigated cerebrospinal fluid (CSF) oxidative stress markers 8-oxo-7,8-dihydroguanosine (8-oxoGuo) and 8-oxo-7,8-dihydro-2′-deoxyguanosine (8-oxodG) that relate to RNA and DNA damage, respectively. Patients with BD (*n* = 86, 51% female) and gender-and-age-matched healthy control individuals (HC; *n* = 44, 44% female) were evaluated at baseline (T0), during (T1) and after a new affective episode (T2), if it occurred, and after a year (T3). Cerebrospinal and urine oxidative stress markers were analyzed using ultra-performance liquid chromatography–tandem mass spectrometry. CSF-8-oxoGuo was statistically significantly higher by 18% (*p* = 0.003) in BD versus HC at T0, and by 22% (*p* *=* 0) at T3. CSF-8-oxoGuo had increased by 15% (*p* = 0.042) from T0 to T3, and by 14% (*p* = 0.021) from T2 to T3 in patients, who experienced an episode during follow-up. CSF-8-oxodG had increased by 26% (*p* = 0.054) from T0 to T2 and decreased by 19% (*p* = 0.041) from T2 to T3 in patients, who experienced an episode during follow-up. CSF-8-oxoGuo did not show a statistically significant change in HC during the one-year follow-up. CSF and urine-8-oxoGuo levels correlated moderately. In conclusion, CSF oxidative stress marker of RNA damage 8-oxoGuo showed both state and trait dependence in BD and stability in HC. Central RNA damage may be a potential biomarker for BD.

## Introduction

Bipolar disorder (BD) is a disabling mental illness with a prevalence of 1%, a high risk of recurrence of manic and depressive episodes, a lifelong elevated risk of suicide^1^ and a heritability of 60–80%^2^. A vast body of literature evidence show clinical progression in BD with increasing risk of developing new mood episodes with every episode, progressive shortening of inter-episode intervals with each recurrence, and with increasing cognitive disabilities during the course of illness^1,3–8^. However, systematic research of the underlying neurobiology of illness progression is lacking.

Elevated levels of peripheral markers of oxidative stress have been found in psychiatric disorders, diabetes, and neurodegenerative disorders^9–11^. Oxidative stress reflects an increase in pro-oxidants, which subsequently leads to oxidative modifications of cellular components, such as RNA and DNA^12^. Oxidative stress markers 8-oxo-7,8-dihydroguanosine (8-oxoGuo), a marker of RNA oxidation, and 8-oxo-7,8dihydro-2′-deoxyguanosine (8-oxodG), a marker of DNA oxidation, can reliably be quantified in cerebrospinal fluid (CSF)^13^ and urine^14^ using a modified ultra-performance liquid chromatography and mass spectrometry assay, and are valid markers of central/whole-body RNA and DNA damage, respectively^14^.

We and other groups have found elevated levels of urine-8-oxoGuo and 8-oxodG in patients with BD compared to healthy control individuals (HC)^9,15–17^. Furthermore, in a longitudinal study our group has found increased oxidative stress in manic/hypomanic states versus remission^17^. Postmortem measurements indicate DNA as the main site of oxidative stress modifications in the central nervous system in severe mental illnesses and suggested that 8-oxoGuo may pass the blood–brain barrier more readily than 8-oxodG^18^. Data are largely missing on oxidative stress evolution during progression of BD^11^ and as recently reviewed, CSF oxidative stress has not yet been investigated in either BD or HC ^19^.

This study aimed, as the first, to investigate state-specific, intra-individual changes in repeated measures of cerebrospinal and urinary markers of oxidative stress in outpatients diagnosed with BD compared to HC individuals during a one-year prospective, longitudinal follow-up study.

The following hypotheses were tested: Cerebrospinal and urinary oxidative stress marker levels are: (1) higher in patients with BD compared to HC, (2) stable during a year in HC, (3) increased during and following an affective episode, and (4) correlated.

## Participants and methods

### Setting

The study was conducted at the Copenhagen Affective Disorder Research Center. Participants for the study were investigated from 1 April 2014 until 27 April 2017. All participants were assessed at baseline (T0) and after a follow-up of one year (T3). The mood states of patients with BD were evaluated by weekly contacts. In case of a new affective episode of depression, hypomania or mania patients were reassessed during the episode (T1) and at the time they had regained remission (T2; Table 1).

**Table 1:** Clinical characteristics of patients with bipolar disorder and healthy control individuals.

	BD baseline (T0)	HC baseline (T0)	*p* value	BD after an episode (T2)	BD follow-up (T3)	HC follow-up (T3)	*p* value
*N* (% female)	86 (51)	44 (54)	0.581^a^	32 (50)	73 (52)	41 (53)	0.846^a^
Age, median (Q1; Q3)	33 (25; 42)	31 (24; 41)	0.526^b^	35 (25; 41)	35 (26; 42)	30 (24; 40)	0.352^b^
Bipolar type I, *N* (%)	49 (57)			18 (56)	43 (58)		
Bipolar type II, *N* (%)	37 (43)			14 (44)	31 (42)		
Clinical Global Impression, mean (s.d.)	4.6 (0.6)			4.6 (0.6)	4.8 (0.6)		
Duration of illness, *N* years, mean (s.d.)	12.4 (9.8)						
Prior psychosis, *N* (%)	36 (41)			13 (41)	31 (42)		
First and second-degree family members with affective disorder, *N* (mean)	2.25 (1.9)	0					
Young mania rating scale, median (Q1; Q3)	1 (0; 2)	0 (0; 0)	<0.001	0 (0; 1)	0 (0; 1)	0 (0; 0)	<0.001
Hamilton depression rating scale 17 items	3 (1; 5)	0 (0; 0)	<0.001	3 (1; 6)	2 (0; 4)	0 (0; 0)	<0.001
Daily alcohol consumption, median (Q1; Q3)	0.2 (0; 1)	0.5 (0; 1)	0.375^b^	0.1 (0; 0.5)	0.3 (0.1; 1)	1 (0.3; 1.2)	0.015^c^
Smokers, *N* (%)	28 (34)	8 (18)	0.096^a^	14 (42)	25 (34)	8 (20)	0.132^a^
Daily cigarettes, median (min; max)	14.5 (0.2; 20)	3.5 (0.5; 30)	0.026^b^	16 (5; 20)	12 (0.5; 35)	2.5 (0.1; 20)	0.032^b^
BMI, mean (s.d.)	25.3 (4.9)	24.9 (3.4)	0.659	24.9. (3.9)	25.6 (5.2)	25.6 (3.7)	0.973
Lithium, *N* (%)	44 (51)			18 (56)	40 (54)		
Antipsychotics, *N* (%)	33 (38)			18 (56)	25 (34)		
Anticonvulsants, *N* (%)	43 (50)			22 (69)	37 (50)		
Antidepressants, *N* (%)	2 (2)			1 (3)	4 (5)		
Benzodiazepines, *N* (%)	6 (7)			7 (22)	9 (12)		
CSF 8-oxoGuo (pmol/L), median (Q1; Q3)	54.8 (47.3; 68)	48.1 (39.3; 56.4)	<0.001		61.5 (54.6; 2.4)	51.8 (41.2; 58.8)	<0.001
Urine 8-oxoGuo (nmol/mmol creatinine), median (Q1; Q3)	1.8 (1.5; 2.1)	1.5 (1.3; 1.8)	<0.001		1.6 (1.4; 2)	1.2 (1.1; 1.5)	<0.001
CSF 8-oxodG (pmol/L) median (Q1; Q3)	6.5 (4.6; 8.9)	5.4 (3.1; 7)	0.016		6.9 (5.8; 8.2)	5.9 (4.5; 7.3)	0.196
Urine 8-oxodG (nmol/mmol creatinine), median (Q1; Q3)	1.4 (1.1; 1.7)	1.3 (1; 1.4)	0.069		1.3 (1.1; 1.6)	1.1 (0.9; 1.3)	<0.001

Summary statistics are *N* (%) for categorical data, mean (s.d.) for normally distributed continuous data and median (lower quartile; upper quartile) for non-normally distributed continuous data

*BD* patients with bipolar disorder, *HC* healthy control individuals

^a^Fisher’s exact test; ^b^data log-transformed, *p* values are *t*-tests of medians are the same; ^c^“Wilcoxon rank” test

All participants provided written informed consent and were reimbursed regarding lumbar puncture.

### Participants

#### Patients with BD

Newly diagnosed patients aged 18–60 years with BD in remission were recruited from the Copenhagen Affective Disorder Clinic that receives patients from the Capital Region of Denmark covering 1.6 million people and all psychiatric centres in the region^20^. Diagnoses were initially provided by experienced psychiatrists in the Clinic. Exclusion criteria were significant physical illness, pregnancy or planned pregnancy within a year, substance abuse, expected noncompliance with the protocol, no informed consent, and finally practical reasons.

#### Healthy control individuals

Age-and-gender-matched HC with no personal or first-degree family history of psychiatric disorders were recruited among blood donors aged 18–60 years affiliated to the Blood Bank at Frederiksberg Hospital, Copenhagen as in prior studies from our group^21^. Exclusion criteria were the same as for the patients.

### Clinical assessment

#### Baseline T0

Written and oral information of the study was given to patients with BD at the Copenhagen Affective Disorder Clinic and at the Blood Bank for the HC followed up by a personal contact by e-mail or telephone. After giving informed consent, the participants were examined at baseline (T0). The clinical diagnosis was evaluated using the semistructured Schedules for Clinical Assessment in Neuropsychiatry (SCAN) interview^22^ conducted by specialist in psychiatry (U.K.). The severity of mood symptoms was assessed using the 17-item Hamilton Depression Rating Scale (HAMD)^23^ and the Young Mania Rating Scale (YMRS)^24^. Remission was defined as scores below 8 on both scales for at least two weeks. Furthermore, clinical characteristics were assessed, including weight, height, current medication, alcohol consumption, smoking habits, duration of illness from first hypomanic episode, and history of psychoses. Severity of illness was estimated using the Global Clinical Impression Scale^25^.

#### Follow-up T1, T2, and T3

**Fig. 1:**
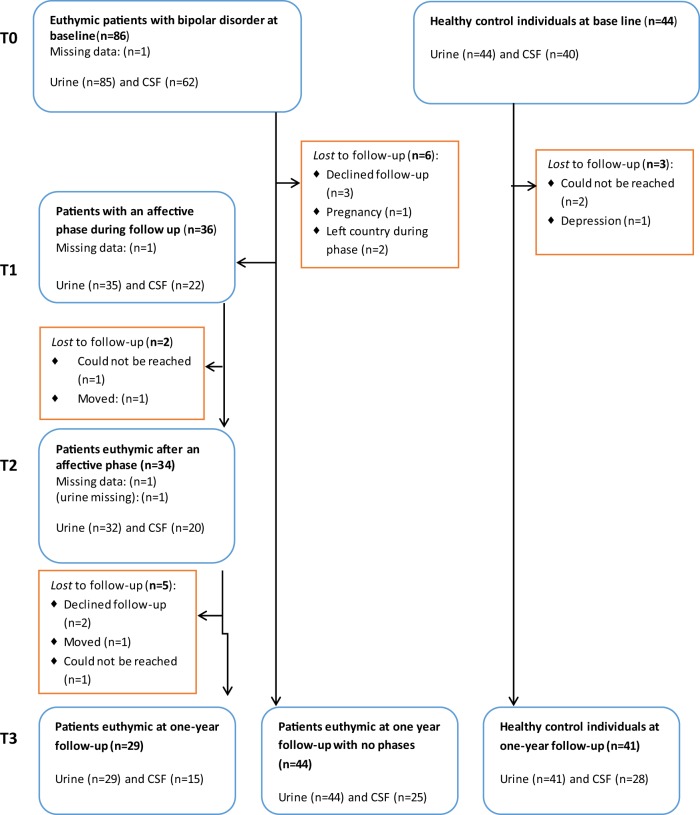
Flowchart for the Bipolar Oxidative Stress Follow-up Study.

All participants were followed prospectively for a year. The patients received treatment as usual and were instructed to daily self-monitoring of mood, sleep, alcohol, and medicine intake. Psychiatrist U.K. kept in weekly contact with the patients by their choices of either telephone, short message service, or e-mail. Patients who experienced a moderate to severe affective episode defined as scores above 13 points on either the HAMD or the YMRS for at least two weeks, had a repeated clinical assessment, including urine, blood, and CSF sampling during the episode (T1) and, also following the episode when being in stable remission for at least two weeks (T2). Finally, all participants were assessed at the one-year follow-up in remission, defined as at least eight weeks in a stable remission state (T3), see Flowchart, Fig. 1. On the basis of prior data from the Copenhagen Affective Disorder Clinic^26^, we expected that 50% of the patients would experience an affective episode during the follow-up period.

### Biological assessments

The participants (fasted overnight before the collection of CSF, blood, and urine samples between 0800 and 1000 h in the morning. At all timepoints (T0, T1, T2, and T3), the clinical assessments and the urine, blood, and CSF sampling from the participants were done on the same or the following day.

#### Sampling and handling of CSF

Specialists of neurology (S.G.H. and P.R.) performed lumbar puncture to collect CSF samples from patients with BD and HC individuals in the lateral decubitus position. The spinal needle was inserted into the L3/L4 or L4/L5 interspace, and a total volume of 10–12 ml of CSF was collected in polypropylene tubes, and gently inverted to avoid gradient effects. Samples where centrifuged on acquisition at 2,000 *g* for 10 min at +4 °C and stored in polypropylene tubes in 250 µL aliquots at −80 °C pending analysis. A general CSF screen was conducted, including albumin, immunoglobulin G (IgG), IgG index, erythrocytes, white blood cells, glucose, and protein.

#### Blood sampling

Board-certified laboratory technicians collected blood samples that were analyzed at the Clinical Biochemical Laboratory at Rigshospitalet, Denmark, regarding standard biochemical parameters, including hematological parameters, blood glucose, C-reactive protein, thyroid hormones, lipid status, ions, metabolites, liver enzymes, and lithium levels.

#### Urine sampling

A freshly voided spot urine was obtained using a standard sampling kit without any additives. The sample was kept on ice and centrifuged at 4 °C and 1590 *g* for 15 min, after which aliquots of 1.5 ml were transferred to Eppendorf tubes and stored at −80 °C pending analyses. The results for oxidative stress markers in urine were normalized for creatine^27^.

#### Analyses of 8-oxoGuo and 8-oxodG

The cerebrospinal and urinary oxidative stress markers 8-oxoGuo and 8-oxodG were analyzed at Laboratory of Clinical Pharmacology, Rigshospitalet using ultra-performance liquid chromatography–tandem mass spectrometry, as described in full detail elsewhere ^13,27^.

### Statistical analyses

Data were analyzed according to a preestablished protocol. All analyses were conducted with SAS software, version 9.4, (Copyright 2013, SAS Institute Inc., Cary, NC, USA). All *p* values were corrected for multiple testing using the Benjamini & Hochberg procedure^28^. We applied a conservative cutoff for the false discovery rate at 0.05, which limits the rate of false positives among the reported findings to one in 20, so that an adjusted *p* ≤ 0.05 was considered statistically significant. All biomarkers were found to have a skew distribution and were therefore log-transformed prior to analysis. Hence, estimated differences between groups and timepoints are expressed in relative terms as percent-wise differences.

Regarding sample size and power, the numbers of participants in this present study, including 86 patients with BD and 44 HC individuals are like the largest prior case-control studies, regarding peripheral oxidative stress markers in BD^15–17^.

#### Demographic and clinical data

Demographic and clinical data at timepoints T0, T2, and T3 were summarized in numbers and percentages (categorical data), means and s.d. (normally distributed continuous data), and medians and quartiles (non-normally distributed continuous data). Comparisons of BD and HC at T0 and T3 was made using Fisher’s exact test, Welch’ *t*-test or the Mann–Whitney *U* test, whichever was most appropriate.

#### Markers of oxidative stress in BD and HC at baseline and at the one-year follow-up

To compare biomarker levels of CSF-8-oxoGuo, CSF-8-oxodG, urine-8-oxoGuo, and urine-8-oxodG between BD and HC, a linear mixed model was applied with time (T0 or T3) and group (BD or HC) as fixed effects and with an unstructured covariance to account for correlation between the repeated measurements on the study participants. The analyses were performed in three versions, version 1: no adjustment for potential confounders; version 2: adjusted for gender, age, and body mass index (BMI); version 3: adjusted for gender, age, BMI, alcohol consumption, and smoking. Estimated differences between BD and HC are reported for biomarker levels at T0, biomarker levels at T3, and change in biomarker level from T0 to T3.

The analyses were repeated with further stratification of BD into the participants who either had or had not experienced an episode during follow-up.

Internal validity of the measured biomarkers was evaluated by comparing their levels at baseline and follow-up in HC.

#### Patients with BD, who had an affective episode during follow-up

A subgroup analysis was performed to evaluate changes in biomarker levels in patients with BD, who had experienced an affective episode during follow-up. To this end, a linear mixed model with timepoint (T0, T1, T2, T3) as fixed effect and an unstructured covariance was applied. Estimates were reported for changes between the timepoints. The analysis was performed in two versions, version 1: no adjustment for potential confounders; and version 2: adjusted for gender, age, BMI, and the three mood stabilizers lithium, quetiapine, and lamotrigine.

#### Correlations between CSF and urinary measures of oxidative stress

Spearman and Pearson correlations were estimated between CSF and urine 8-oxoGuo and 8-oxodG at timepoint T0 and T3, and in BP and HC separately.

#### Effect of dose of lithium, quetiapine, lamotrigine and smoking on CSF and urinary measures of oxidative stress

To investigate the effect of the three mood stabilizers, we applied a linear mixed model as previously described^29^, which distinguishes the cross-sectional effect (i.e., the effect of the average dose of the drug over time) from the longitudinal effect (i.e., the effect of changes in the dose of the drug over time) in order to address potential biases due to unmeasured confounders. In mixed models, the effect of smoking on each of the four outcomes was estimated with inclusion of data from all four timepoints.

#### Sensitivity analyses

All analyses were repeated including and excluding outliers. This did not alter the results to any significant extent. We report data including outliers.

## Results

### Inclusion, demographics, clinical, and study characteristics

Out of a total of 497 eligible patients with BD, 86 patients were included in the study in remission. A total of 411 did not enter the study due to: not obtaining remission before the inclusion ended in January 2016 (*n* = 57), significant physical illness (*n* = 97), pregnancy or planned pregnancy (*n* = 62), substance abuse (*n* = 26), expected noncompliance with the protocol (*n* = 90), not giving informed consent (*n* = 49), and discharge from the clinic before an informed consent could be obtained (*n* = 30).

Demographics and clinical characteristics of the participants of the study are presented in Table 1. A total of 24 participants (BD = 15, HC = 9) received medical treatment for a stabilized physical disorder or as hormone anticonception: hypertension (BD = 1), diabetes mellitus type II (BD = 1), hypothyroidism (BD = 3, HC = 1), and hormonal contraceptives (BD = 10, HC = 9). Patients were most frequently treated with lithium, lamotrigine, and quetiapine, but three patients did not get any psychotropic medication at inclusion.

A total of 44 HC were included in the study. Patients with BD and HC individuals were well matched according to age, gender and, BMI at baseline and there were no differences either at follow-up. There were more smokers among patients with BD at baseline and at follow-up alcohol intake was higher in HC individuals.

The flow chart (Fig. 1) shows that 36 patients with BD developed a new affective episode during follow-up and of these 34 reached stable remission within the study period. The completion rates from baseline to follow-up for patients with BD and HC were 65% versus 86% regarding CSF, and 70% versus 93% regarding urine samples. All together 62 patients with BD and 40 HC gave samples of both CSF and urine at baseline. All participants, but one, provided a urine sample at baseline (BD = 85, HC = 44).

### Levels of cerebrospinal and urinary oxidative stress marker levels in patients with BD compared to HC

CSF-8-oxoGuo was statistically significantly higher by 18% (95% confidence interval (CI) 8–28%, adj-*p* = 0.003) in patients with BD versus HC at baseline, and by 22% (95% CI 12–34%, adj-*p* = 0) at follow-up.

Urine-8-oxoGuo was statistically significantly higher by 17% (95% CI 8–27%, adj-*p* = 0.003) in patients with BD versus HC at baseline, and by 30% (95% CI 16–45%, adj-*p* = 0) at follow-up.

**Fig. 2:**
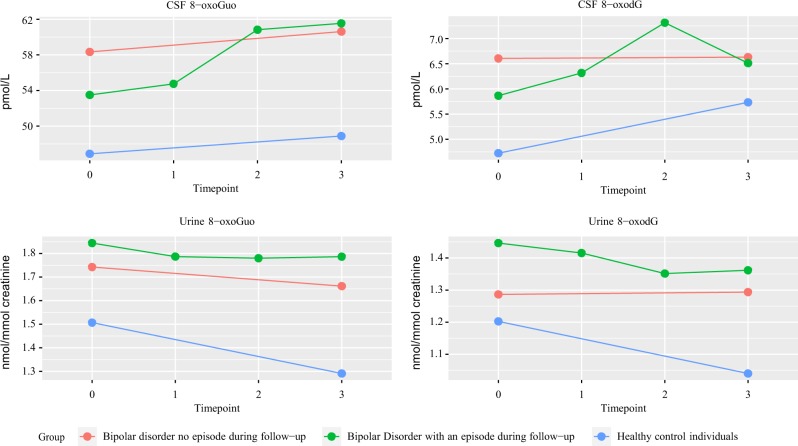
Cerebrospinal fluid and urine levels of 8-oxoGuo and 8-oxodG in patients with bipolar disorder with and without an episode during follow-up and healthy control individuals at baseline (T0), during (T1), and after (T2) an episode, did it occur and, at one-year follow-up.

CSF-8-oxodG was statistically significantly higher by 29% (95% CI 6–55%, adj-*p* = 0.043) at baseline, and urine-8-oxodG was statistically significantly higher by 25% (95% CI 10–43%, adj-*p* = 0.005) at follow-up in BD versus HC. CSF-8-oxodG was higher by 13% at follow-up and urine-8-oxodG was higher by 12% at baseline in patients with BD versus HC, but these differences were not statistically significant in the adjusted models (Fig. 2 and Table 2).

**Table 2:** Increased levels of cerebrospinal and urinary oxidative stress markers 8-oxoGuo and 8-oxodG in patients with bipolar disorder compared to healthy control individuals at baseline and at one-year follow-up.

Outcome	*b*	Version 1*, b1*	*p* value (adj.)	Lower	Upper	Version 2, *b2*	*p* value (adj.)	Lower	Upper	Version 3, *b3*	*p* value (adj.)	Lower	Upper
CSF 8-oxoGuo	T0 BD/HC	1.20	0.001 (0.005)	1.08	1.33	1.19	<0.001 (0.001)	1.09	1.29	1.18	<0.001 (0.003)	1.08	1.28
	T3 BD/HC	1.24	<0.001 (0.002)	1.11	1.38	1.24	<0.001 (<0.001)	1.13	1.35	1.22	0 < 0.001 (<0.001)	1.12	1.34
Urine 8-oxoGuo	T0 BD/HC	1.19	<0.001 (0.001)	1.10	1.30	1.18	<0.001 (0.001)	1.09	1.29	1.17	<0.001 (0.003)	1.08	1.27
	T3 BD/HC	1.33	<0.001 (<0.001)	1.19	1.48	1.31	<0.001 (<0.001)	1.18	1.45	1.30	<0.001 (<0.001)	1.16	1.45
CSF 8-oxodG	T0 BD/HC	1.33	0.006 (0.03)	1.09	1.62	1.33	0.003 (0.017)	1.11	1.60	1.29	0.01 (0.043)	1.06	1.55
	T3 BD/HC	1.16	0.092 (0.218)	0.98	1.38	1.17	0.049 (0.14)	1.00	1.37	1.13	0.149 (0.298)	0.96	1.32
Urine 8-oxodG	T0 BD/HC	1.13	0.04 (0.119)	1.01	1.28	1.14	0.031 (0.101)	1.01	1.29	1.12	0.074 (0.193)	0.99	1.27
	T3 BD/HC	1.28	<0.001 (0.001)	1.13	1.45	1.27	<0.001 (0.002)	1.12	1.44	1.25	0.001 (0.005)	1.10	1.43

Estimated differences between BD and HC are reported for biomarker levels at T0, biomarker levels at T3, and change in biomarker level from T0 to T3. Version 1 is uncorrected; version 2 is corrected for gender, age, and body mass index; and version 3 is corrected for gender, age, body mass index, smoking, and alcohol consumption. The number in any of the *b*-columns is an estimate of how much larger the median outcome is among BD relative to HC

*BD* patients with bipolar disorder, *adj*. adjusted, *HC* healthy control individuals

When patients with BD were divided into subgroups with and without a new affective episode during follow-up, no statistically significant differences in the oxidative stress marker levels were found between the subgroups at the timepoints baseline (T0) and follow-up (T3) (Supplemental Table 1). Furthermore, when considering the relative changes from T0 to T3 in the subgroups with and without an episode during follow-up compared to HC, no differences were found in the relative changes between T0 and T3 (Supplemental Table 2).

Regarding the internal validity of the biomarkers only CSF-8-oxoGuo did not show a statistically significant change in HC during the one-year follow-up (Supplemental Table 3). On the contrary, CSF-8-oxodG in HC increased significantly by 22% while urine-8-oxoGuo and urine-8-oxodG decreased significantly by 15 and 14%, respectively (Supplemental Table 3).

### Changes in levels of cerebrospinal and urinary oxidative stress marker levels in patients with BD during and following an affective episode

CSF-8-oxoGuo had increased by 15% (95% CI 4–27%, adj-*p* = 0.042) from T0 to T3, and by 14% (95% CI 6–23%, adj-*p* = 0.021) from T1 (during an episode) to T3 in patients who experienced an episode during follow-up (Table 3).

**Table 3:** Changes in cerebrospinal fluid and urinary oxidative stress markers levels of 8-oxoGuo and 8-oxodG in patients with bipolar disorder who had an affective episode during a one-year follow-up.

Outcome	*b*	Version 1*, b1*	*p* value (adj.)	Lower	Upper	Version 2, *b2*	*p* value (adj.)	Lower	Upper
CSF 8-oxoGuo	T3/T0	1.16	0.004 (0.023)	1.05	1.27	1.15	0.01 (0.042)	1.04	1.27
	T2/T0	1.12	0.027 (0.094)	1.01	1.23	1.09	0.131 (0.0275)	0.97	1.22
	T1/T0	1.02	0.512 (0.661)	0.96	1.09	1.01	0.815 (0.898)	0.93	1.09
	T3/T1	1.13	0.004 (0.021)	1.05	1.22	1.14	0.004 (0.021)	1.06	1.23
	T2/T1	1.09	0.059 (0.158)	1.00	1.20	1.08	0.116 (0.252)	0.98	1.19
	T3/T2	1.03	0.477 (0.634)	0.94	1.14	1.06	0.27 (0.435)	0.95	1.17
Urine 8-oxoGuo	T3/T0	0.97	0.588 (0.735)	0.86	1.09	0.92	0.162 (0.316)	0.82	1.03
	T2/T0	0.97	0.506 (0.655)	0.87	1.07	0.95	0.43 (0.586)	0.85	1.07
	T1/T0	0.97	0.409 (0.569)	0.90	1.05	0.94	0.199 (0.352)	0.86	1.03
	T3/T1	1.00	0.991 (0.997)	0.90	1.11	0.98	0.669 (0.807)	0.87	1.09
	T2/T1	1.00	0.943 (0.98)	0.90	1.10	1.01	0.829 (0.909)	0.91	1.12
	T3/T2	1.00	0.949 (0.98)	0.92	1.10	0.97	0.362 (0.522)	0.89	1.04
CSF 8-oxodG	T3/T0	1.05	0.5 (0.655)	0.90	1.23	1.02	0.757 (0.871)	0.88	1.19
	T2/T0	1.25	0.001 (0.008)	1.10	1.41	1.26	0.014 (0.054)	1.05	1.51
	T1/T0	1.05	0.565 (0.712)	0.89	1.22	1.09	0.322 (0.486)	0.92	1.28
	T3/T1	1.01	0.915 (0.977)	0.86	1.18	0.94	0.114 (0.249)	0.87	1.02
	T2/T1	1.19	0.045 (0.133)	1.00	1.42	1.16	0.127 (0.273)	0.95	1.41
	T3/T2	0.85	0.038 (0.116)	0.72	0.99	0.81	0.01 (0.042)	0.70	0.94
Urine 8-oxodG	T3/T0	0.96	0.4 (0.56)	0.87	1.06	0.94	0.182 (0.332)	0.85	1.03
	T2/T0	0.93	0.134 (0.278)	0.84	1.02	0.89	0.03 (0.1)	0.81	0.99
	T1/T0	0.99	0.88 (0.954)	0.90	1.09	0.97	0.482 (0.638)	0.98	1.07
	T3/T1	0.97	0.474 (0.634)	0.87	1.07	0.97	0.562 (0.711)	0.87	1.08
	T2/T1	0.93	0.19 (0.341)	0.84	1.04	0.92	0.165 (0.317)	0.82	1.04
	T3/T2	1.03	0.4 (0.56)	0.96	1.12	1.05	0.222 (0.379)	0.97	1.14

Estimates *b* were reported for changes between the timepoints: T1–T0, T2–T0, T3–T0, T2–T1, T3–T1, and T3–T2. The analysis was performed in two versions. Version 1: no adjustment for potential confounders; and version 2: adjusted for gender, age, BMI, and the three mood stabilizers lithium, quetiapine, and lamotrigine. *p* values corrected for multiple testing are in brackets.

*T0* baseline, *T1* during an episode, *T2* after an episode, *T3* at one-year follow-up, *adj*. adjusted

CSF-8-oxodG had increased by 26% (95% CI 5–51%, adj-*p* = 0.054) from T0 to T2 (after an episode) and decreased by 19% (95% CI −30–6%, adj-*p* = 0.041) from T2 to T3 in patients who experienced an episode during follow-up.

No statistically significant changes were found in the other CSF and urinary oxidative stress markers or between any of the remaining timepoints (Table 3).

### Correlations between cerebrospinal and urinary oxidative stress markers

Measures of cerebrospinal and urinary oxidative stress markers of nucleoside damage correlated in separate analyses of all participants, patients with BD and HC (Supplemental Table 4).

Strong statistically significant correlations were found between CSF-8-oxoGuo and CSF-8-oxodG in patients, with BD and HC individuals at both baseline and follow-up. Weak to moderate statistically significant correlations were found between CSF-8-oxoGuo and urine-8-oxoGuo in patients, with BD and HC individuals at both baseline and follow-up. Furthermore, moderate statistically significant correlations were found between CSF-8-oxodG and urine-8-oxodG in patients, with BD and HC individuals at baseline and follow-up regarding HC individuals. However, a weak correlation between CSF-8-oxodG and urine-8-oxodG was not statistically significant in patients with BD at follow-up (Supplemental Table 4).

### The influence of medication on oxidative stress markers

Measures of cerebrospinal and urinary oxidative stress markers of nucleoside damage tended to increase with increasing doses of lithium. The longitudinal effect, i.e., the effect of individual changes in dose of lithium was the strongest on CSF-8-oxoGuo (+0.9% per mmol increase in dose, 95% CI 0.3–1.6%, *p*-adj = 0.04) and remained statistically significant in multivariate analysis and after adjustment for multiple testing. A similar effect was only borderline statistically significant on CSF-8-oxodG (+1.2% per mmol increase in dose, 95% CI 0.3–2.2%, *p*-adj = 0.06). No significant effects of increasing doses of quetiapine and lamotrigine were found in 8oxoGuo and 8oxodG in either CSF or urine (Supplemental Table 5).

### The influence of other covariates on oxidative stress markers in patients with BD

As predicted, measures of cerebrospinal and urinary oxidative stress markers of nucleoside damage depend on age and gender. However, there was no general effect of other covariates, including smoking and alcohol (Supplemental Table 6).

## Discussion

This study confirmed that levels of CSF-8-oxoGuo: (1) were statistically significantly higher at both baseline and follow-up in patients with BD compared to HC, (2) showed internal validity since the values in HC did not change from baseline to follow-up, (3) increased following an affective episode in patients with BD, and (4) correlated moderately with levels of urine-8-oxoGuo. Thus, cerebrospinal oxidative stress markers of RNA damage 8-oxoGuo showed both state and trait dependence in BD and stability in HC. In subgroup comparisons between patients with BD either with or without an episode during follow-up, a new affective episode was not predictable from baseline levels of oxidative stress markers.

Explorative analyses showed that increasing doses of lithium were associated with an increase of cerebrospinal and urinary oxidative stress markers, but this may likely be a result of confounding by indication (higher doses of lithium prescribed for more severe BDs). Furthermore, prior studies suggested that lithium was associated with decreased peripheral oxidative stress marker levels in euthymic patients with BD^30–32^.

This study found different pathophysiological characteristics in centrally and systemically generated oxidative stress. The results suggest that a possible pathogenic effect may be found down-stream from DNA, since no statistically significant differences between BD and HC were found regarding DNA damage measured by 8-oxodG, but merely in 8-oxoGuo that represents RNA damage. Also, the only weak to moderate correlation between centrally and systemically generated oxidative stress emphasize a role of the blood–brain barrier.

The findings regarding urinary oxidative stress from the present study are consistent with prior findings from our group, showing increased levels of urine markers of oxidative stress in unipolar depression^33^, BD rapid cycling^16^, BD type I^17^, and schizophrenia^34^. In these prior studies, state dependencies were found only in patients diagnosed with BD type I between the states of mania and stable remission^17^.

Increased levels of oxidative stress may predict mortality in patients with other chronic diseases such as diabetes^35^. Epidemiological studies have found that the life expectancy among patients with BD is decreased by 8–12 years compared to the general population^36^ and that patients die due to natural causes of death already from adolescence^37^. It is a possibility that increased levels of oxidative stress seen in patients with BD may contribute to the decreased life expectancy by inducing accelerated ageing. The present results suggest that relapses of affective episodes may increase oxidative stress. This gives hope that effective long-term preventive treatment may contribute to normalized life expectancy in BD. Furthermore, our findings suggest that CSF oxidative stress may represent state (increased at T1) and trait markers (increased at T2 and T3) in BD, and may reflect neurobiological correlates of illness progression and sensitization^5,38^ in BD.

Mitochondrial dysfunction reflected as increased oxidative stress may be a biological underpinning of BD^11,39^, and impaired autophagy has been suggested as the link between mitochondrial dysfunction and psychiatric disorders^11^.

Overall, our findings show that central RNA damage may be related to the pathogenesis of BD.

## Limitations

Only a total of 36 patients (42%) experienced an affective episode during the follow-up period and the subjects had fewer repeated CSF samples than urine samples. Data were analyzed using linear mixed models, which implicitly imputes missing data from missed samples and drop outs, and provides unbiased results under the assumption that missing data is missing at random. However, results may still be biased if missingness depends on confounding factors that are not accounted for. Smoking was more prevalent in BD compared to HC. However, analyses of the effect of smoking showed no significant effect in uni- and mulitivariate analyses. Estimates and *p* values remained significant after adjusting for alcohol and smoking. Clinical researchers (U.K., S.G.H., and P.R.) were not blinded to the participant being BD or HC. However, they did not participate in the statistical analyses of the oxidative stress markers that for all practical reasons were blinded.

## Supplementary information

Supplemetatry Tables 1–6
