# Supplementary material for: Cerebrospinal fluid oxidative stress metabolites in patients with bipolar disorder and healthy controls: a longitudinal case-control study
Source: Transl Psychiatry. 2019 Nov 28;9:325. doi: 10.1038/s41398-019-0664-6 (PMC6882849; doi:10.1038/s41398-019-0664-6)
Supplement: Supplementary file 1 — Supplemetatry Tables 1–6 [file 41398_2019_664_MOESM1_ESM.docx]

Supplementary Table 1. Cerebrospinal fluid and urinary oxidative stress markers 8-oxoGuo and 8-oxodG in subgroups of patients with bipolar disorder with and without an affective episode during follow-up compared to healthy control individuals at baseline and follow-up after one year

| Outcome | *b* | Version 1*, b1* | p-value (adj.) | Lower | Upper |  | Version 2, *b2* | p-value (adj.) | Lower | Upper | Version 3, *b3* | p-value (adj.) | Lower | Upper |
| --- | --- | --- | --- | --- | --- | --- | --- | --- | --- | --- | --- | --- | --- | --- |
| CSF  8-oxoGuo | T0 NE/HC | 1.24 | 0.001 (0.004) | 1.10 | 1.40 |  | 1.20 | <0.001  (0.004) | 1.08 | 1.32 | 1.19 | 0.001  (0.005) | 1.08 | 1.31 |
|  | T0 E/HC | 1.14 | 0.032 (0.103) | 1.01 | 1.29 |  | 1.17 | 0.002  (0.014) | 1.06 | 1.28 | 1.14 | 0.011  (0.045) | 1.03 | 1.27 |
|  | T0 E/NE | 0.92 | 0.183 (0.332) | 0.81 | 1.04 |  | 0.97 | 0.613  (0.754) | 0.88 | 1.08 | 0.96 | 0.468  (0.628) | 0.86 | 1.07 |
|  | T3 NE/HC | 1.24 | 0.001  (0.005) | 1.10 | 1.40 |  | 1.20 | <0.001  (0.004) | 1.09 | 1.32 | 1.19 | 0.001  (0.004) | 1.08 | 1.32 |
|  | T3 E/HC | 1.25 | 0.001 (0.007) | 1.10 | 1.42 |  | 1.30 | <0.001  (<0.001) | 1.17 | 1.45 | 1.28 | <0.001  (0.001) | 1.14 | 1.43 |
|  | T3 E/NE | 1.01 | 0.922  (0.98) | 0.88 | 1.15 |  | 1.08 | 0.157  (0.308) | 0.97 | 1.22 | 1.07 | 0.254  (0.417) | 0.95 | 1.20 |
| Urine  8-oxoGuo | T0 NE/HC | 1.16 | 0.004 (0.021) | 1.05 | 1.27 |  | 1.14 | 0.005  (0.025) | 1.04 | 1.25 | 1.14 | 0.008  (0.038) | 1.03 | 1.25 |
|  | T0 E/HC | 1.22 | <0.001  (0.002) | 1.11 | 1.36 |  | 1.23 | <0.001  (0.001) | 1.11 | 1.35 | 1.21 | <0.001  (0.003) | 1.09 | 1.34 |
|  | T0 E/NE | 1.06 | 0.269 (0.435) | 0.96 | 1.17 |  | 1.07 | 0.151  (0.299) | 0.97 | 1.18 | 1.06 | 0.203  (0.353) | 0.97 | 1.17 |
|  | T3 NE/HC | 1.29 | <0.001  (0.001) | 1.14 | 1.45 |  | 1.26 | <0.001  (0.002) | 1.12 | 1.41 | 1.25 | <0.001  (0.003) | 1.11 | 1.41 |
|  | T3 E/HC | 1.38 | <0.001  (<0.001) | 1.21 | 1.57 |  | 1.38 | <0.001  (<0.001) | 1.21 | 1.57 | 1.36 | <0.001  (<0.001) | 1.19 | 1.56 |
|  | T3 E/NE | 1.07 | 0.294  (0.456) | 0.94 | 1.22 |  | 1.10 | 0.156  (0.307) | 0.96 | 1.25 | 1.09 | 0.190  (0.341) | 0.96 | 1.24 |
| CSF  8-oxodG | T0 NE/HC | 1.40 | 0.006  (0.030) | 1.10 | 1.77 |  | 1.35 | 0.008  (0.038) | 1.08 | 1.69 | 1.33 | 0.013  (0.050) | 1.06 | 1.66 |
|  | T0 E/HC | 1.24 | 0.072  (0.191) | 0.98 | 1.57 |  | 1.29 | 0.022  (0.084) | 1.04 | 1.61 | 1.22 | 0.088  (0.211) | 0.97 | 1.53 |
|  | T0 E/NE | 0.89 | 0.351  (0.515) | 0.69 | 1.14 |  | 0.96 | 0.707  (0.828) | 0.76 | 1.21 | 0.92 | 0.477  (0.634) | 0.72 | 1.16 |
|  | T3 NE/HC | 1.16 | 0.150  (0.298) | 0.95 | 1.41 |  | 1.11 | 0.254  (0.417) | 0.93 | 1.32 | 1.09 | 0.339  (0.505) | 0.91 | 1.30 |
|  | T3 E/HC | 1.17 | 0.146  (0.293) | 0.94 | 1.46 |  | 1.28 | 0.015  (0.058) | 1.05 | 1.56 | 1.19 | 0.100  (0.230) | 0.97 | 1.47 |
|  | T3 E/NE | 1.02 | 0.890  (0.959) | 0.81 | 1.27 |  | 1.16 | 0.165  (0.317) | 0.94 | 1.42 | 1.09 | 0.415  (0.574) | 0.88 | 1.36 |
| Urine  8-oxodG | T0 NE/HC | 1.07 | 0.330  (0.497) | 0.93 | 1.23 |  | 1.08 | 0.292  (0.456) | 0.94 | 1.23 | 1.06 | 0.405  (0.565) | 0.92 | 1.22 |
|  | T0 E/HC | 1.20 | 0.012  (0.048) | 1.04 | 1.39 |  | 1.21 | 0.009  (0.039) | 1.05 | 1.40 | 1.18 | 0.029  (0.098) | 1.02 | 1.37 |
|  | T0 E/NE | 1.12 | 0.105  (0.237) | 0.98 | 1.30 |  | 1.13 | 0.097  (0.227) | 0.98 | 1.30 | 1.11 | 0.142  (0.289) | 0.96 | 1.28 |
|  | T3 NE/HC | 1.24 | 0.002  (0.014) | 1.08 | 1.43 |  | 1.22 | 0.007  (0.034) | 1.06 | 1.40 | 1.20 | 0.011  (0.046) | 1.04 | 1.39 |
|  | T3 E/HC | 1.32 | <0.001  (0.004) | 1.13 | 1.53 |  | 1.33 | <0.001  (0.003) | 1.14 | 1.55 | 1.30 | 0.001  (0.008) | 1.11 | 1.53 |
|  | T3 E/NE | 1.06 | 0.447  (0.605) | 0.91 | 1.23 |  | 1.10 | 0.240  (0.401) | 0.94 | 1.28 | 1.08 | 0.312  (0.478) | 0.93 | 1.26 |

Footnotes: Abbreviations: HC: healthy control individual; NE: Patient with bipolar disorder with no episode; E: Patient with bipolar disorder with an episode during follow-up. Version 1 is un-corrected, Version 2 is corrected for gender, age, body mass index, Version 3 is corrected for gender, age, body mass index, smoking and, alcohol consumption. An example of the interpretation of a row in the table: ‘T0 NE/HC’ concerns healthy controls and patients without episode at T0. Because the outcome is log-transformed, the interpretation is the following: the number in any of the b-columns is an estimate of [the median of the outcome among NE’s] divided by [the median of the outcome among HC’s] at T0. I.e.it is an estimate of “how much larger the outcome is for NE’s relative to the HC’s”. If the estimate is above 1, the median among NE’s are higher than the median among HC’s. P-values corrected for multiple testing are in brackets.

Supplementary Table 2. Relative changes from baseline to follow-up in cerebrospinal fluid and urinary oxidative stress markers 8-oxoGuo and 8-oxodG in patients with bipolar disorder with and without an affective episode during follow-up compared to healthy control individuals

| Outcome | *b* | Version 1*, b1* | p-value (adj.) | Lower | Upper | Version  2, *b2* | p-value (adj.) | Lower | Upper | | Version  3, *b3* | | p-value (adj.) | Lower | Upper |
| --- | --- | --- | --- | --- | --- | --- | --- | --- | --- | --- | --- | --- | --- | --- | --- |
| CSF 8-oxoGuo | (NE T3/T0) /  (HC T3/T0) | 1.00 | 0.934 (0.980) | 0.92 | 1.08 | 1.00 | 0.961  (0.986) | 0.92 | 1.09 | 1.00 | | 0.954  (0.980) | | 0.92 | 1.09 |
|  | (E T3/T0) /  (HC T3/T0) | 1.09 | 0.053 (0.145) | 1.00 | 1.20 | 1.12 | 0.024  (0.088) | 1.01 | 1.23 | 1.11 | | 0.028  (0.096) | | 1.01 | 1.23 |
|  | (E T3/T0) /  (NE T3/T0) | 1.10 | 0.052  (0.143) | 1.00 | 1.21 | 1.11 | 0.033  (0.104) | 1.01 | 1.23 | 1.11 | | 0.039  (0.119) | | 1.01 | 1.23 |
| Urine 8-oxoGuo | (NE T3/T0) /  (HC T3/T0) | 1.09 | 0.167 (0.317) | 0.96 | 1.23 | 1.07 | 0.251  (0.417) | 0.95 | 1.21 | 1.08 | | 0.234  (0.395) | | 0.95 | 1.21 |
|  | (E T3/T0) /  (HC T3/T0) | 1.13 | 0.076  (0.195) | 0.99 | 1.28 | 1.12 | 0.086  (0.208) | 0.98 | 1.28 | 1.13 | | 0.077  (0.195) | | 0.99 | 1.28 |
|  | (E T3/T0) /  (NE T3/T0) | 1.03 | 0.604  (0.748) | 0.91 | 1.18 | 1.05 | 0.502  (0.655) | 0.92 | 1.19 | 1.05 | | 0.491  (0.647) | | 0.92 | 1.19 |
| CSF 8-oxodG | (NE T3/T0) /  (HC T3/T0) | 0.83 | 0.039  (0.119) | 0.69 | 0.99 | 0.82 | 0.031  (0.101) | 0.69 | 0.98 | 0.82 | | 0.030  (0.100) | | 0.69 | 0.98 |
|  | (E T3/T0) /  (HC T3/T0) | 0.88 | 0.218  (0.375) | 0.72 | 1.08 | 0.92 | 0.426  (0.583) | 0.76 | 1.12 | 0.92 | | 0.393  (0.556) | | 0.75 | 1.12 |
|  | (E T3/T0) /  (NE T3/T0) | 1.07 | 0.527  (0.676) | 0.87 | 1.31 | 1.13 | 0.255  (0.417) | 0.92 | 1.38 | 1.12 | | 0.296  (0.456) | | 0.91 | 1.38 |
| Urine 8oxodG | (NE T3/T0) /  (HC T3/T0) | 1.16 | 0.020  (0.074) | 1.03 | 1.32 | 1.13 | 0.048  (0.140) | 1.00 | 1.28 | 1.14 | | 0.041  (0.121) | | 1.01 | 1.29 |
|  | (E T3/T0) /  (HC T3/T0) | 1.12 | 0.106  (0.238) | 0.98 | 1.29 | 1.13 | 0.086  (0.208) | 0.98 | 1.29 | 1.13 | | 0.076  (0.195) | | 0.99 | 1.30 |
|  | (E T3/T0) /  (NE T3/T0) | 0.96 | 0.611  (0.754) | 0.84 | 1.11 | 1.00 | 0.946  (0.980) | 0.87 | 1.14 | 0.99 | | 0.941  (0.980) | | 0.87 | 1.14 |

Footnotes: Abbreviations: HC: healthy control individual; NE: No episode; E: Episode. Version 1 is un-corrected, Version 2 is corrected for gender, age, body mass index, Version 3 is corrected for gender, age, body mass index, smoking and, alcohol consumption. An example of the interpretation of a row: ‘(NE T3/T0)/ (HC T3/T0)’ concerns the HC’s and NE’s at T0 and T3. Because the outcome is log-transformed, the interpretation is the following: the number in any of the b-columns is an estimate of, how much larger the median outcome among NE’s is at T3 relative to T0, divided by how much larger the median outcome among HC’s is at T3 relative to T0. I.e. it is an estimate of how much larger the rise in outcome-level is among NE’s relative to among HC’s. P-values corrected for multiple testing are in brackets.

| Outcome | *b* | Version 1*, b1* | p-value (adj.) | Lower | Upper | Version 2, *b2* | p-value (adj.) | Lower | Upper |
| --- | --- | --- | --- | --- | --- | --- | --- | --- | --- |
| CSF 8-oxoGuo | T3/T0 | 1.04 | 0.060  (0.160) | 1.00 | 1.09 | 1.04 | 0.086  (0.208) | 0.99 | 1.09 |
| Urine 8-oxoGuo | T3/T0 | 0.86 | <0.001  (0.001) | 0.80 | 0.92 | 0.85 | <0.001  (0.001) | 0.79 | 0.92 |
| CSF 8-oxodG | T3/T0 | 1.21 | 0.008  (0.037) | 1.05 | 1.39 | 1.22 | 0.009  (0.041) | 1.05 | 1.42 |
| Urine 8-oxodG | T3/T0 | 0.86 | <0.001  (0.001) | 0.80 | 0.92 | 0.86 | <0.001  (0.002) | 0.80 | 0.93 |

Supplementary Table 3. Internal validity of cerebrospinal fluid and urinary oxidative stress markers 8-oxoGuo and 8-oxodG in healthy individuals during a one-year follow-up

Footnotes: Estimates of levels of oxidative stress markers 8-oxoGuo and 8-oxodG (medians) in healthy control individuals at baseline T0 versus one-year follow-up T3. An estimate >1 means that the level was higher at T3 compared to T0). Version 1 is un-corrected, Version 2 is corrected for gender, age, body mass index, smoking and alcohol consumption. P-values corrected for multiple testing are in brackets.

Supplemental Table 4. Spearman and Pearson correlations between cerebrospinal fluid and urinary oxidative stress markers 8-oxoGuo and 8-oxodG in patients with bipolar disorder versus healthy control individuals at baseline and follow-up

| Outcome 1 | Outcome 2 | Baseline | | | | | Follow-up | | | | |
| --- | --- | --- | --- | --- | --- | --- | --- | --- | --- | --- | --- |
| Patients with bipolar disorder | | Spearman-correl. | Pearson-  correl. | | p-value  (adjusted) | Confidence-interval | Spearman-correl. | Pearson-  correl. | | p-value  (adjusted) | Confidence-interval |
| CSF 8-oxoGuo | CSF 8-oxodG | 0.72 | | 0.77 | <0.001 (<0.001) | 0.64, 0.85 | 0.64 | | 0.82 | <0.001 (<0.001) | 0.69, 0.9 |
| CSF 8-oxoGuo | Urine 8-oxoGuo | 0.63 | | 0.64 | <0.001 (<0.001) | 0.47, 0.77 | 0.51 | | 0.4 | 0.010  (0.05) | 0.1, 0.63 |
| CSF 8-oxoGuo | Urine 8-oxodG | 0.25 | | 0.25 | 0.050  (0.14) | 0, 0.47 | 0.13 | | 0.16 | 0.340  (0.5) | -0.16, 0.45 |
| CSF 8-oxodG | Urine 8-oxodG | 0.61 | | 0.57 | <0.001 (<0.001) | 0.37, 0.72 | 0.37 | | 0.26 | 0.100  (0.23) | -0.05, 0.53 |
| CSF 8-oxodG | Urine 8-oxoGuo | 0.54 | | 0.53 | <0.001 (<0.001) | 0.32, 0.69 | 0.23 | | 0.21 | 0.200  (0.35) | -0.11, 0.49 |
| Urine 8-oxoGuo | Urine 8-oxodG | 0.6 | | 0.59 | <0.001 (<0.001) | 0.43, 0.71 | 0.59 | | 0.63 | <0.001 (<0.001) | 0.47, 0.75 |
| Healthy control individuals | |  | | | | |  | | | | |
| CSF 8-oxoGuo | CSF 8-oxodG | 0.65 | | 0.59 | <0.001  (<0.001) | 0.34, 0.76 | 0.74 | | 0.64 | <0.001 (<0.001) | 0.34, 0.82 |
| CSF 8-oxoGuo | Urine 8-oxoGuo | 0.51 | | 0.63 | <0.001 (<0.001) | 0.34, 0.78 | 0.31 | | 0.48 | 0.001  (0.04) | 0.13, 0.72 |
| CSF 8-oxoGuo | Urine 8-oxodG | 0.37 | | 0.36 | 0.020  (0.09) | 0.05, 0.6 | 0.09 | | 0.1 | 0.620  (0.76) | -0.29, 0.45 |
| CSF 8-oxodG | Urine 8-oxodG | 0.51 | | 0.55 | <0.001 (<0.001) | 0.29, 0.74 | 0.39 | | 0.43 | 0.020  (0.09) | 0.06, 0.69 |
| CSF 8-oxodG | Urine 8-oxoGuo | 0.18 | | 0.19 | 0.230  (0.39) | -0.13, 0.48 | 0.14 | | 0.11 | 0.590  (0.74) | -0.28, 0.46 |
| Urine 8-oxoGuo | Urine 8-oxodG | 0.59 | | 0.56 | <0.001 (<0.001) | 0.31, 0.73 | 0.57 | | 0.48 | <0.001 (0.01) | 0.2, 0.69 |

Footnotes: The p-values are a test of the hypothesis that the correlation is zero. The intervals of confidence are at 95% levels. The oxidative stress markers correlations are calculated from log-transformed measures of oxidative stress marker levels. P-values corrected for multiple testing are in brackets.

Supplementary Table 5. The effects of dose of lithium (LI), quetiapine (AP), and lamotrigine (AC) on cerebrospinal fluid and urinary oxidative stress 8-oxoGuo and 8-oxodG in patients with bipolar disorder versus healthy control individuals at baseline and follow-up

Univariate models

| Compound | Outcome | Cross-sectional effect  estimate | Longitudinal effect  estimate | p-value  cross sectional | p-value  longitudinal | p-value  difference |
| --- | --- | --- | --- | --- | --- | --- |
| AC | CSF 8-oxoGuo | 1.049 (1.004;1.097) | 1.041 (0.991;1.093) | 0.03 (0.11) | 0.11 (0.24) | 0.80 (0.89) |
|  | Urine 8-oxoGuo | 1.005 (0.971;1.041) | 0.989 (0.931;1.05) | 0.77 (0.88) | 0.71 (0.83) | 0.65 (0.78) |
|  | CSF 8-oxodG | 1.111 (1.032;1.196) | 1.035 (0.961;1.115) | 0.01 (0.03) | 0.36 (0.52) | 0.18 (0.33) |
|  | Urine 8-oxodG | 0.998 (0.948;1.052) | 0.985 (0.92; 1.055) | 0.95 (0.98) | 0.66 (0.80) | 0.76 (0.87) |
| AP | CSF 8-oxoGuo | 0.993 (0.948;1.041) | 1.029 (0.995;1.109) | 0.77 (0.88) | 0.45 (0.60) | 0.42 (0.58) |
|  | Urine 8-oxoGuo | 1.014 (0.984;1.046) | 1.086 (0.982;1.202) | 0.36 (0.52) | 0.11 (0.24) | 0.21 (0.37) |
|  | CSF 8-oxodG | 0.993 (0.948;1.041) | 1.029 (0.955;1.109) | 0.77 (0.88) | 0.45 (0.60) | 0.42 (0.58) |
|  | Urine 8-oxodG | 0.991 (0.944;1.042) | 0.978 (0.88;1.087) | 0.73 (0.85) | 0.67 (0.81) | 0.82 (0.90) |
| LI | CSF 8-oxoGuo | 1.002 (0.997;1.007) | 1.009 (1.004;1.014) | 0.42 (0.58) | <0.001 0 (0.01) | 0.05 (0.14) |
|  | Urine 8-oxoGuo | 1.005 (1.001;1.008) | 1.007 (1.001;1.013) | 0.02 (0.06) | 0.03 (0.09) | 0.50 (0.65) |
|  | CSF 8-oxodG | 0.999 (0.991;1.007) | 1.014 (1.007;1.022) | 0.76 (0.87) | <0.001 (<0.001) | 0.01 (0.03) |
|  | Urine 8-oxodG | 1.003 (0.997;1.008) | 1.01 (1.003;1.016) | 0.37 (0.53) | <0.001 (0.02) | 0.09 (0.22) |

Multivariate models

| Compound | Outcome | Cross-sectional effect estimate | Longitudinal effect estimate | p-value  cross sectional | p-value  longitudinal | p-value  difference |
| --- | --- | --- | --- | --- | --- | --- |
| AC | CSF 8-oxoGuo | 1.051 (0.983;1.124) | 1.049 (0.985;1.117) | 0.14 (0.29) | 0.13 (0.28) | 0.97 (0.99) |
|  | Urine 8-oxoGuo | 1.045 (0.991;1.101) | 0.983 (0.901;1.073) | 0.10 (0.23) | 0.70 (0.82) | 0.23 (0.39) |
|  | CSF 8-oxodG | 1.117 (0.966;1.291) | 1.096 (1.001;1.199) | 0.13 (0.27) | 0.05 (0.14) | 0.82 (0.9) |
|  | Urine 8-oxodG | 1 (0.907;1.102) | 0.978 (0.89;1.075) | 1.00 (1.00) | 0.65 (0.78) | 0.75 (0.86) |
| AP | CSF 8-oxoGuo | 0.973 (0.918;1.031) | 1.015 (0.942;1.093) | 0.34 (0.50) | 0.69 (0.82) | 0.35 (0.52) |
|  | Urine 8-oxoGuo | 0.966 (0.929;1.004) | 1.069 (0.964;1.185) | 0.08 (0.20) | 0.20 (0.35) | 0.07 (0.19) |
|  | CSF 8-oxodG | 0.984 (0.868);1.116) | 0.986 (0.888;1.095) | 0.80 (0.89) | 0.79 (0.89) | 0.98 (0.99) |
|  | Urine 8-oxodG | 0.951 (0.885;1.023) | 0.968 (0872;1.075) | 0.17 (0.32) | 0.53 (0.68) | 0.78 (0.88) |
| LI | CSF 8-oxoGuo | 1.004 (0.997;1.011) | 1.009 (1.003;1.016) | 0.27 (0.44) | 0.01 (0.04) | 0.28 (0.45) |
|  | Urine 8-oxoGuo | 1.004 (0.998;1.009) | 1.004 (0.996;1.011) | 0.17 (0.32) | 0.32 (0.48) | 1.00 (1.00) |
|  | CSF 8-oxodG | 1.004 (0.989;1.02) | 1.012 (1.003;1.022) | 0.57 (0.72) | 0.01 (0.06) | 0.39 (0.56) |
|  | Urine 8-oxodG | 0.999 (0.989;1.009) | 1.008 (1;1.016) | 0.89 (0.96) | 0.05 (0.14) | 0.18 (0.33) |

Footnotes: The analysis was performed in two versions. Univariate models 1: No adjustment for potential confounders, Multivariate models: adjusted for gender, age, BMI, bipolar type I or II, prior psychosis, and global clinical impression. Note: the doses for lamotrigine and quetiapine were scaled by 1/100 so that an increase by a single unit could have a measurable impact. Thus, the dose-units were mmol for lithium and 100 mg for both lamotrigine and quetiapine.

Supplemental Table 6, Effects of co-variates on measures of cerebrospinal and urine oxidative stress in 86 patients with bipolar disorder

|  | CSF 8-oxoGuo |  |  |  | Urine 8-oxoGuo |  |  |  |
| --- | --- | --- | --- | --- | --- | --- | --- | --- |
| Covariate | *b* | p-value (adj.) | Lower | Upper | *b* | p-value (adj.) | Lower | Upper |
| Gender | 1.220 | 0.001 (0.007) | 1.093 | 1.363 | 1.074 | 0.103 (0.329) | 0.985 | 1.170 |
| BD I/II | 0.954 | 0.499 (0.751) | 0.829 | 1.097 | 0.947 | 0.371 (0.672) | 0.838 | 1.069 |
| AC | 1.184 | <0.001 (0.005) | 1.079 | 1.298 | 1.102 | 0.034 (0.142) | 1.007 | 1.206 |
| AP | 1.015 | 0.679 (0.841) | 0.945 | 1.090 | 1.034 | 0.360 (0.667) | 0.962 | 1.112 |
| LI | 1.118 | 0.022 (0.100) | 1.017 | 1.230 | 1.127 | 0.010 (0.061) | 1.029 | 1.235 |
| Prior  psychosis | 1.039 | 0.594 (0.822) | 0.901 | 1.197 | 1.148 | 0.024 (0.101) | 1.019 | 1.293 |
| Age | 1.014 | <0.001 (0.002) | 1.007 | 1.021 | 1.006 | 0.039 (0.149) | 1.000 | 1.012 |
| BMI | 1.009 | 0.085 (0.285) | 0.999 | 1.020 | 1.003 | 0.436 (0.724) | 0.995 | 1.012 |
| Global  Clinical Impression | 0.995 | 0.155 (0.410) | 0.988 | 1.002 | 0.999 | 0.787 (0.874) | 0.994 | 1.005 |
| Duration  of illness | 1.000 | 0.932 (0.960) | 0.999 | 1.001 | 1.000 | 0.295 (0.586) | 1.000 | 1.001 |
| Smoking | 0.999 | 0.787 (0.874) | 0.993 | 1.005 | 1.001 | 0.646 (0.838) | 0.996 | 1.006 |
| Alcohol | 0.972 | 0.284 (0.578) | 0.923 | 1.024 | 1.003 | 0.699 (0.844) | 0.986 | 1.021 |

|  | CSF 8-oxodG |  |  |  | Urine 8-oxodG |  |  |  |
| --- | --- | --- | --- | --- | --- | --- | --- | --- |
| Covariate | *b* | p-value (adj.) | Lower | Upper | *b* | p-value (adj.) | Lower | Upper |
| Gender | 1.360 | 0.006 (0.039) | 1.098 | 1.684 | 1.095 | 0.226 (0.535) | 0.944 | 1.269 |
| BD I/II | 0.943 | 0.655 (0.838) | 0.724 | 1.228 | 0.935 | 0.520 (0.775) | 0.759 | 1.151 |
| AC | 1.188 | 0.018 (0.081) | 1.032 | 1.368 | 1.059 | 0.366 (0.669) | 0.935 | 1.198 |
| AP | 0.955 | 0.216 (0.518) | 0.881 | 1.035 | 1.006 | 0.896 (0.937) | 0.913 | 1.109 |
| LI | 1.178 | 0.012 (0.064) | 1.053 | 1.317 | 1.172 | 0.011 (0.063) | 1.038 | 1.324 |
| Prior  Psychosis | 0.923 | 0.558 (0.809) | 0.701 | 1.215 | 1.157 | 0.159 (0.412) | 0.943 | 1.419 |
| Age | 1.023 | 0.001 (0.006) | 1.010 | 1.036 | 1.001 | 0.856 (0.913) | 0.991 | 1.011 |
| BMI | 1.022 | 0.012 (0.063) | 1.007 | 1.038 | 0.992 | 0.274 (0.573) | 0.979 | 1.006 |
| Global  Clinical Impression | 0.991 | 0.181 (0.441) | 0.978 | 1.004 | 0.994 | 0.244 (0.547) | 0.985 | 1.004 |
| Duration  of illness | 0.999 | 0.407 (0.699) | 0.998 | 1.001 | 1.000 | 0.818 (0.886) | 0.999 | 1.001 |
| Smoking | 1.003 | 0.526 (0.778) | 0.991 | 1.016 | 1.001 | 0.770 (0.837) | 0.993 | 1.009 |
| Alcohol | 0.948 | 0.259 (0.559) | 0.862 | 1.042 | 1.014 | 0.173 (0.439) | 0.993 | 1.036 |

Footnotes: Abbreviations: BMI: Body Mass Index, BD I/II: bipolar type I or II. Regarding the binary outcomes the estimate refer to male, BP I, medication with Lithium (LI), anticonvulsant lamotrigine (AC) and antipsychotic quetiapine (AP), and prior psychosis. P-values corrected for multiple testing are in brackets.
